# Matrix- and plasma-derived peptides promote tissue-specific injury responses and wound healing in diabetic swine

**DOI:** 10.1186/s12967-016-0946-1

**Published:** 2016-07-02

**Authors:** Anthony R. Sheets, Conner J. Massey, Stephen M. Cronk, Mark D. Iafrati, Ira M. Herman

**Affiliations:** Graduate Program in Cellular & Molecular Physiology, The Sackler School of Graduate Biomedical Sciences, Tufts University, Boston, MA 02111 USA; Graduate Program in Cell, Molecular and Developmental Biology, The Sackler School of Graduate Biomedical Sciences, Tufts University, Boston, MA 02111 USA; Department of Developmental, Molecular and Chemical Biology, School of Medicine, Tufts University, 136 Harrison Ave, Boston, MA 02111 USA; The Center for Innovations in Wound Healing Research, School of Medicine, Tufts University, 136 Harrison Ave, Boston, MA 02111 USA; Department of Surgery, Division of Vascular Surgery, Tufts Medical Center, 800 Washington St., Boston, MA 02111 USA

**Keywords:** Diabetic foot ulcer, Angiogenesis, Re-epithelialization, Molecular medicine, Chronic wound care

## Abstract

**Background:**

Non-healing wounds are a major global health concern and account for the majority of non-traumatic limb amputations worldwide. However, compared to standard care practices, few advanced therapeutics effectively resolve these injuries stemming from cardiovascular disease, aging, and diabetes-related vasculopathies. While matrix turnover is disrupted in these injuries, debriding enzymes may promote healing by releasing matrix fragments that induce cell migration, proliferation, and morphogenesis, and plasma products may also stimulate these processes. Thus, we created matrix- and plasma-derived peptides, Comb1 and UN3, which induce cellular injury responses in vitro, and accelerate healing in rodent models of non-healing wounds. However, the effects of these peptides in non-healing wounds in diabetes are not known. Here, we interrogated whether these peptides stimulate healing in a diabetic porcine model highly reminiscent of human healing impairments in type 1 and type 2-diabetes.

**Methods:**

After 3–6 weeks of streptozotocin-induced diabetes, full-thickness wounds were surgically created on the backs of adult female Yorkshire swine under general anesthesia. Comb1 and UN3 peptides or sterile saline (negative control) were administered to wounds daily for 3–7 days. Following sacrifice, wound tissues were harvested, and quantitative histological and immunohistochemical analyses were performed for wound closure, angiogenesis and granulation tissue deposition, along with quantitative molecular analyses of factors critical for angiogenesis, epithelialization, and dermal matrix remodeling.

**Results:**

Comb1 and UN3 significantly increase re-epithelialization and angiogenesis in diabetic porcine wounds, compared to saline-treated controls. Additionally, fluorescein-conjugated Comb1 labels keratinocytes, fibroblasts, and vascular endothelial cells in porcine wounds, and Far western blotting reveals these cell populations express multiple fluorescein-Comb1-interacting proteins in vitro. Further, peptide treatment increases mRNA expression of several pro-angiogenic, epithelializing, and matrix-remodeling factors, importantly including balanced inductions in matrix metalloproteinase-2, -9, and tissue inhibitor of metalloproteinases-1, lending further insight into their mechanisms.

**Conclusions:**

Comb1 and UN3 stimulate wound resolution in diabetic Yorkshire swine through upregulation of multiple reparative growth factors and cytokines, especially matrix metalloproteinases and inhibitors that may aid in reversing the proteolytic imbalance characteristic of chronically inflamed non-healing wounds. Together, these peptides should have great therapeutic potential for all patients in need of healing, regardless of injury etiology.

## Background

Non-healing wounds associated with cardiovascular disease, aging, and diabetes are a major global healthcare issue. The number of patients with diabetes worldwide is anticipated to exceed 400 million by 2025 [1], and at least 15 % of these patients will develop diabetic foot ulcers (DFU) during their lifetime [2]. These wounds directly precede 80 % of nontraumatic limb amputations, globally [3], with an annual mortality estimated to be greater than that of several major cancers [4]. The greatest increases in diabetes are expected in regions with limited healthcare access; here, patients may have less glycemic control due to the expense of insulin and other medicines, precipitating higher rates of peripheral neuropathy, DFU, and ultimately amputation [2]. Moreover, despite the predicted rise in DFU prevalence, along with cardiovascular disease- and aging-associated non-healing wounds, there remain few advanced therapeutics that effectively improve healing outcomes compared to standard care practices, including debridement and mechanical off-loading [5]. Thus, there is a vastly unmet clinical need for safe, effective, and affordable chronic wound treatments across the globe.

The modest outcomes observed following treatment with current molecular medicines may stem from the inflamed, protease-rich DFU microenvironment [6], which deprives cells of growth factors and matrix-derived signals necessary for tissue repair [7, 8] and may degrade exogenously applied growth factor-based therapeutics such as becaplermin [9]. Hence, molecular interventions that resist proteolytic cleavage, as well as normalize the chronic wound protease-to-inhibitor ratio, are innovative strategies that may resolve DFU. Intriguingly, matrix-remodeling enzymes such as *Clostridial* collagenase, and matrix-derived preparations such as platelet-rich plasma (PRP), each have been demonstrated to induce tissue repair in DFU [10, 11], suggesting these entities promote healing through mechanisms unimpeded by the chronic wound protease burden. Thus, in our efforts to uncover these mechanisms and develop advanced therapeutics, we identified bioactive peptides liberated from *Clostridial* collagenase-digested vascular endothelial extracellular matrix (ECM) and thrombin-derived peptides in PRP that stimulate cell migration, proliferation, and microvascular morphogenesis [12, 13]. In turn, we created short, protease-resistant, combinatorial peptides comprised of the individual ECM (Comb1) and PRP peptides (UN3), which further augment endothelial cell proliferation and morphogenesis, and post-injury keratinocyte migration in vitro [12, 13]. Moreover, each of these combinatorial peptides enhances wound vascularization, granulation tissue formation, and re-epithelialization in cyclophosphamide-treated, healing-impaired mice, and wounds treated with Comb1 and UN3, together, display greater angiogenesis and a thicker epidermis than those treated with either peptide alone [13].

Based on these results, we hypothesized Comb1 and UN3 would induce healing in diabetic wounds. Here, we demonstrate Comb1 and UN3 stimulate post-injury angiogenesis, granulation tissue formation, and re-epithelialization in a pre-clinical swine model of impaired healing, which is highly reminiscent and parallels the human pathophysiologic responses to injury observed in chronically-impaired, non-healing wounds [14]. Additionally, fluorescein isothiocyanate (FITC)-conjugated Comb1 labels cell populations in diabetic porcine wound tissues, including fibroblasts and endothelial cells in granulation tissue and keratinocytes present near the wound edge, as well as several millimeters distal to the injury. Further, FITC-Comb1 interacts with multiple proteins isolated from adult human keratinocytes propagated under low Ca^2+^ conditions and elevated temperature (HaCaT), adult human dermal microvascular endothelial cells (HMVEC), and human foreskin fibroblasts (HFF), with binding patterns dependent on cellular responses to injury in vitro. Finally, Comb1 and UN3 significantly stimulate mRNA expression of pro-angiogenic growth factors and receptors, including vascular endothelial growth factor-A (VEGFA), VEGF receptor 1 and 2 (VEGFR1/2), and fibroblast growth factor-2 (FGF2), immune and stem cell chemoattractants including stromal cell-derived factor 1α (SDF1α) and chemokine (C-X-C motif) receptor-4 (CXCR4), and re-epithelialization factors including epidermal growth factor (EGF), heparin-binding EGF-like (HB-EGF), and EGFR in a time- and tissue-specific pattern in diabetic porcine tissues. Importantly, peptide treatment induces matrix metalloproteinase-2 (MMP2), MMP9, and tissue inhibitor of metalloproteinases-1 (TIMP1) mRNA expression, which may aid in restoring the protease/inhibitor balance to promote healing. Overall, these bioactive peptides may be a novel therapeutic approach to interconvert non-healing DFU into acute, actively healing wounds.

## Methods

### Animal studies

#### Diabetic induction

Adult female Yorkshire swine weighing 50–60 kg were made diabetic by intravenous administration of streptozotocin (STZ, Teva Pharmaceuticals, Petah Tikva, Israel) at a dose of 150 mg/kg as described [15]. Blood glucose levels were obtained twice daily using a OneTouch UltraMini glucometer (LifeScan, Inc., Milpitas, CA), with diabetes confirmed by glucose levels greater than 200 mg/dL; insulin was administered twice daily on a sliding scale to maintain levels between 250 and 600 mg/dL, to model poor glycemic control.

#### Full thickness excisional wounding and post-injury treatments

Following 3–6 weeks of diabetic induction, animals underwent Tufts University IACUC-approved dorsal wounding surgeries: after anesthesia induction with a telazol/ketamine/xylazine cocktail, dorsal hair was removed with depilatory cream, followed by shaving. Under general anesthesia with isoflurane, the paraspinal area was prepared and draped sterilely, and full-thickness (5.0 mm deep) excisional wounds were created in the animals’ backs using sterile, stainless steel biopsy punches (8.0 mm-diameter) or a sterile No. 11 surgical blade (2.0 cm × 2.0 cm square wounds). Following hemostasis by manual compression, wounds were covered using 10 % compound tincture of benzoin adhesive and Tegaderm transparent dressing (3 M, St. Paul, MN) and animals were placed in protective jackets. Comb1 (1.0 mg/mL) and UN3 (284 μg/mL) peptides (synthesized by Anaspec, Fremont, CA) were suspended in autoclaved 4 % carboxymethylcelluose and administered to wounds daily by injection beneath the Tegaderm bandage, for a period of 3–7 days. Sterile saline served as a negative control.

#### Tissue harvesting

Immediately following euthanasia, dorsal wounds, including the underlying subcutaneous tissue and fascia, together with an approximately 1 cm perimeter of uninjured cutaneous tissue bordering the wound, were harvested. Wounds were bisected using a sterile surgical blade. Half of each wound was fixed in buffered 4 % formaldehyde and reserved for histology. A 1 mm-thick section was removed from the remaining half of each wound, partitioned into granulation tissue, hypodermis, and adjacent, non-wounded dermis and epidermis using a sterile, stainless steel razor blade, and each piece of tissue was placed in RNAlater RNA stabilization reagent (Qiagen, Venlo, NL). The remaining tissues were placed in large cryovials, snap-frozen in liquid nitrogen, and reserved for immunohistochemistry.

### Quantitative histology and immunohistochemistry of healing responses

#### Histology, immunohistochemistry, and microscopy of fixed, paraffin-embedded tissues

After fixation in buffered formaldehyde for 72 h, wounds were embedded in paraffin, cut into 5 μm-thick sections, and stained with hematoxylin and eosin (H&E) or Masson’s trichrome. Microscopic imaging of H&E and trichrome-stained sections was performed as described [13].

#### Immunhistochemistry and microscopy of fresh frozen tissues

Flash frozen pieces of diabetic porcine wounds were re-equilibrated in PBS-buffered 0.5 % formaldehyde and 15 % sucrose for 2 days at 4C, followed by 2 days in PBS-buffered 0.5 % formaldehyde and 30 % sucrose, and embedded in Tissue-Tek OCT compound (VWR, Radnor, PA). 18 μm-thick sections were cut and placed on Superfrost Plus glass slides (Fisher Scientific, Pittsburgh, PA). Fluorescent immunohistochemistry using mouse anti-CD31 (1:200, Novus Biologicals, Littleton, CO) and rabbit anti-HSPG (1:200), and subsequent microscopy were performed as described [13].

#### Labeling tissue with FITC-conjugated Comb1

18 μm-thick sections of fresh frozen saline-treated porcine wounds were blocked with PBS containing 5 % normal goat serum (NGS) for 1 h at room temperature in a humidified chamber, then incubated with 100 nM FITC-Comb1 in 5 % NGS, protected from light. Fluorescent immunohistochemistry using rabbit anti-FITC primary antibody (1:100, Life Technologies) and subsequent microscopy were performed using methods described [13]. Slides incubated with 5 % NGS alone served as negative controls.

#### Quantitative analysis of wound healing responses

Composite images of H&E, trichrome, and immuno-labeled wounds were created as described [13]. Percent wound closure was determined by dividing the total linear distance of keratinocyte migration from the wound edge by the total width of each wound, each measured using the NIH ImageJ freehand line tool. Angiogenic responses were quantified from merged immunofluorescence images: total wounded area, defined as the histologically apparent interruption in normal skin morphology, and granulation tissue area, defined as the area with dual positivity for CD31 and HSPG, were quantified using the NIH ImageJ freehand tool, and the percentage of granulation tissue occupying the wound was calculated; microvascular density within the granulation tissue was calculated by measuring CD31/HSPG co-localization intensity using NIH ImageJ [13], and normalizing against the granulation tissue area.

### Peptide binding studies

#### Cell culture

Primary adult HMVEC (Lonza Bioscience, Walkersville, MD) were cultured in Endothelial Basal Medium-2 (Lonza) supplemented with 5 % fetal bovine serum (FBS) and the growth factors contained within the EGM2-MV kit (Lonza), according to the manufacturer’s instructions. HFF and HaCaT (gifts from the laboratory of Dr. Jonathan Garlick, Tufts) were grown in DMEM supplemented with 10 % FBS (Atlanta Biologicals, Atlanta, GA), 1 % antibiotic–antimycotic, 1 % l-glutamine, and 10 mM HEPES (Life Technologies). HMVEC were used at P5–8, HFF were used at P14–22, and HaCaT were used at P40–46.

#### Cellular responses to injury in vitro

Cells were seeded in 6-well plates (VWR) and allowed to grow to confluence. 7–10 days later, concentric scratch wounds were created in post-confluent monolayers using stainless steel rakes that uniformly denude ~50 % of the resting population in each well [16]. Denuded cells (time 0) were collected, lysed in RIPA buffer supplemented with a protease inhibitor cocktail (Sigma-Aldrich, St. Louis, MO), and mechanically disrupted by dounce homogenization on ice; 24 h after injury, the migrating cell populations were lysed and homogenized in the same manner. Equal amounts of protein from these homogenates were mixed with reducing sample buffer containing a final concentration of 2 % β-mercaptoethanol (Sigma-Aldrich), heated to 95 °C for 5 min, and separated by SDS-PAGE.

#### Peptide blot overlay

After SDS-PAGE, protein samples were transferred to Whatman Protran 0.1 μm pore size nitrocellulose membranes (GE Healthcare, Little Chalfont, UK) and blocked for 1 h at room temperature with a protein-free blocking solution (Life Technologies). Membranes were then overlaid with protein-free blocking solution (Life Technologies) containing FITC-conjugated Comb1 peptide (200 nM, synthesized by Biomatik, Cambridge, ON) and incubated for 1 hour at room temperature with end-over-end rotation, protected from light. Membranes were washed three times in PBS, and peptides were cross-linked to putative receptors using 2 mM 1-ethyl-3-(3-dimethylaminopropyl)carbodiimide hydrochloride (EDC, Life Technologies) and 2 mM *N*-hydroxysuccinimide (NHS, Life Technologies) for 15 min at room temperature. Cross-linking was quenched by addition of three volumes of 200 mM glycine, 20 mM Tris–HCl, pH 7.5, for 3 min at room temperature. Membranes were washed in TBS and blocked overnight at 4 °C in protein-free blocking solution. Western blotting was performed using goat anti-FITC (1:100, Life Technologies) overnight at 4 °C, followed by incubation with horseradish peroxidase-conjugated donkey anti-goat secondary antibody (1:2000, Santa Cruz Biotechnology, Santa Cruz, CA), and developed using Clarity ECL western blotting substrate (BioRad, Hercules, CA). Digital imaging of Western blots was performed using a BioSpectrum Imaging System with VisionWorks software (UVP, Upland, CA).

### Quantitative molecular analyses

#### RNA isolation

Total RNA was isolated from compartments of diabetic porcine wounds (adjacent, unwounded dermis and epidermis, hypodermis, and granulation tissue) by homogenization in TRIzol (Life Technologies) and purification with RNEasy mini columns (Qiagen).

#### Quantitative real-time PCR

One microgram RNA was reverse transcribed using the Quantitect Reverse Transcription kit (Qiagen). Reactions containing 5 ng cDNA were prepared in duplicate using Taqman probes and Taqman Gene Expression Master Mix (Life Technologies) according to the manufacturer’s instructions, and PCR was performed using an iCycler iQ5 real-time PCR detection system (BioRad). Relative mRNA expression was determined following normalization to beta-actin or RPL32 using the 2^−∆∆Ct^ method.

### Data analysis and statistics

Excisional wounding experiments were performed a total of seven times; 7-days peptide treatments were performed five times, and 3-days peptide treatments were performed twice. In all quantitative analyses, mean values were compared for each treatment group on day 3 and 7 post-wounding, and all data are expressed as fold change relative to saline controls on each day ± SEM. All statistical analyses were performed using an unpaired Student’s *t* test. Peptide blot overlays were performed at least three times.

## Results

### Comb1 and UN3 enhance wound-healing re-epithelialization and angiogenesis in diabetic swine

**Fig. 1:**
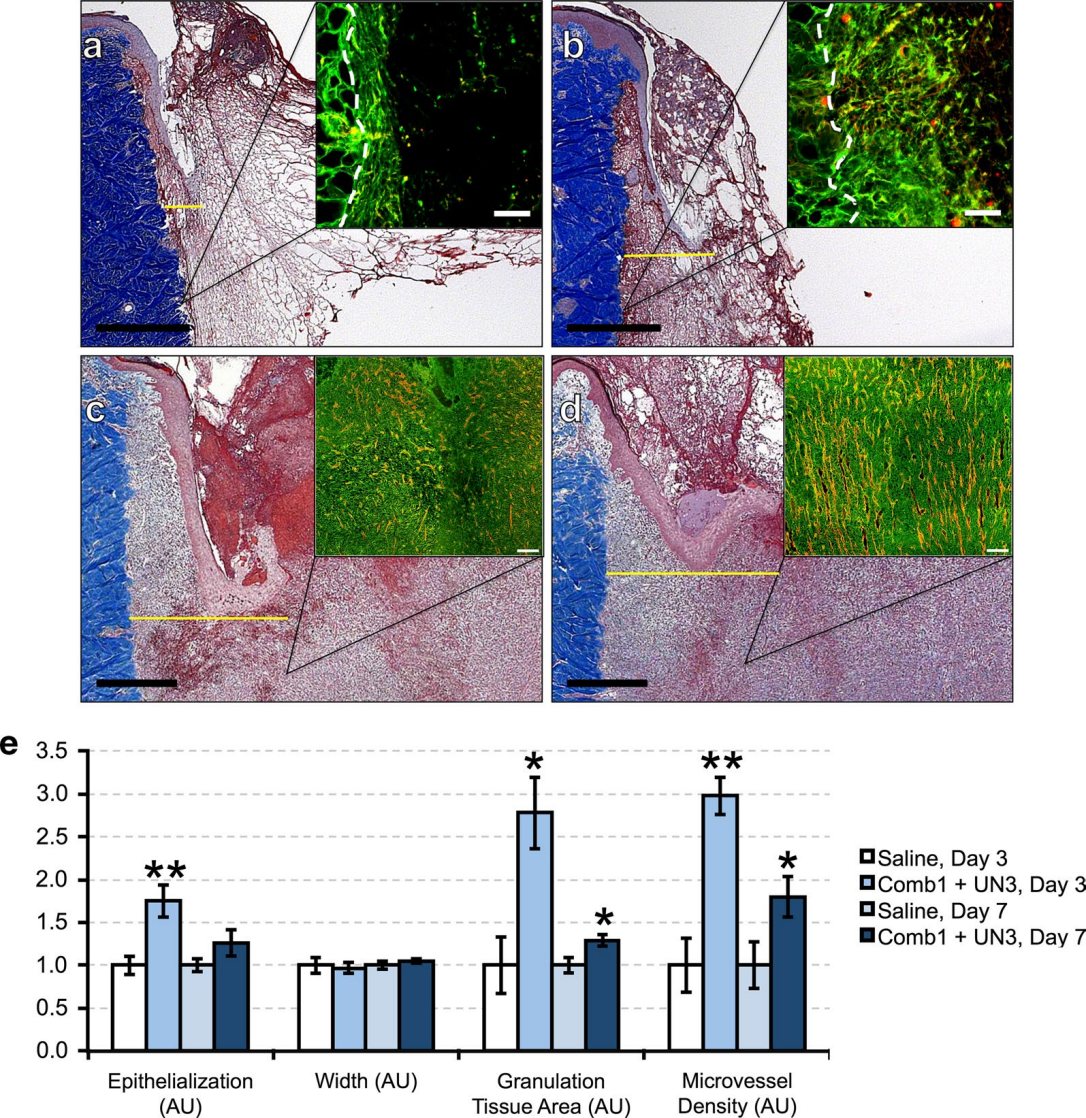
Comb1 and UN3 enhance wound-healing re-epithelialization and angiogenesis in diabetic swine. (**a**–**d**) Representative trichrome staining of wounds treated with saline for 3 days (**a**) or 7 days (**c**) and Comb1 + UN3 (**b**, **d**). *Scale bar* (*black*) denotes 1 mm in all photomicrographs of trichrome-stained tissues, and the *yellow line* denotes the linear distance traversed by migrating keratinocytes. Representative HSPG (*green*) and CD31 (*red*) co-staining for each treatment condition and time point are displayed as insets in* upper right* of each* panel*; *scale bar* (*white*) denotes 100 μm, and the *dashed white line* in the insets of (**a**) and (**b**) denotes the lateral border between injured and non-injured tissue. Data in (**e**) are expressed as mean wound re-epithelialization, width, granulation tissue area, and microvascular density normalized to saline-treated controls on each day, ±SEM, *p ≤ 0.05, **p ≤ 0.01. For re-epithelialization and wound width measurements: Saline day 3, n = 9; Comb1 + UN3 day 3, n = 10; Saline day 7, n = 18; Comb1 + UN3 day 7, n = 24. For granulation tissue area and microvascular density measurements: Saline day 3, n = 3; Comb1 + UN3 day 3, n = 5; Saline day 7, n = 9; Comb1 + UN3 day 7, n = 10

Our past work demonstrates that the matrix- and plasma-derived peptides, Comb1 and UN3, stimulate capillary endothelial cell proliferation and morphogenesis, keratinocyte migration, and produce complete wound closure with highly vascularized granulation tissue in murine models of impaired healing [12, 13]. As a result, we interrogated whether comparable doses of these same peptides induce wound-healing re-epithelialization, angiogenesis, and granulation tissue formation in a diabetic porcine model of impaired healing. Histomorphometric analyses following full thickness excisional wounding reveal that 3 days of peptide treatment (Fig. 1b) significantly increases re-epithelialization by 75 % compared to saline (Fig. 1a, quantified in Fig. 1e). By day 7, peptide-treated wounds (Fig. 1d) demonstrate a 26 % increase in wound closure over controls (Fig. 1c, quantified in Fig. 1e, p = 0.17). Importantly, there is no appreciable difference between the width of peptide- and saline-treated wounds at either time point, suggesting Comb1 and UN3 do not alter wound contraction. Additionally, quantitative immunohistochemistry of heparan sulfate proteoglycan (HPSG) and CD31 in diabetic porcine wounds reveals 3 days of peptide treatment (insets, Fig. 1a, b) significantly triples granulation tissue area and vascular density versus saline-treated controls, and 7 days of peptide treatment (insets, Fig. 1c, d) significantly increases these parameters by 30 and 80 %, respectively (Fig. 1e).

### Comb1 interactions depend on cell type and injury status

**Fig. 2:**
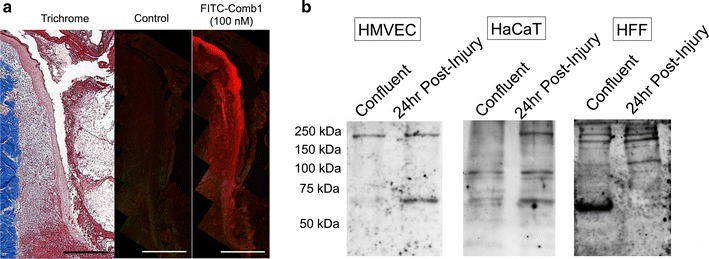
Cytological and biochemical characteristics of FITC-Comb1 interactions. **a** FITC-conjugated Comb1 binds to blood vessels and keratinocytes. Fresh frozen sections from diabetic porcine wounds were blocked with 5 % normal goat serum in PBS, then incubated in block solution alone or containing 100 nM FITC-Comb1, followed by immunofluorescence microscopy using anti-FITC (1:200, Life Technologies). Image analysis reveals that FITC-Comb1 binds to wound-associated keratinocytes, stromal, and vascular cells, as well as keratinocytes distal to the cutaneous defect. Image of trichrome-stained tissue provided for location context. *Scale bar* denotes 500 μm in all panels. **b** FITC-conjugated Comb1 ligates with numerous proteins expressed in HMVEC, HaCaT, and HFF. Whole cell lysates were prepared from cells denuded from post-confluent monolayers, representing the control/resting state of the cells, and actively recovering cells collected 24 h after injury, then separated by SDS-PAGE. Gels were transferred to nitrocellulose membranes, overlaid with 200 nM FITC-Comb1, and cross-linked to putative receptors and interactors using 2 mM EDC and 2 mM NHS. Interactors were detected using by western blotting with an anti-FITC antibody

As our results indicate Comb1, alone, or in combination with UN3, stimulates wound-healing re-epithelialization, angiogenesis, and granulation tissue formation, we interrogated Comb1 localization patterns in diabetic porcine wounds. FITC-Comb1 labels migratory keratinocytes, along with stromal and vascular cells present within granulation tissue at the wound margin in sections of diabetic porcine wounds (Fig. 2a). FITC-Comb1 also labels these cells several millimeters distal to the site of injury. Thus, Comb1 interacts with dermal and epidermal cell populations actively engaged in proliferative and migratory processes, as well as non-injured, non-migratory cells.

Based on these tissue localization patterns, we next investigated whether Comb1 interacts with distinct proteins in post-confluent HMVEC, HaCaT, and HFF, and in migratory cells post-injury. FITC-Comb1 binds multiple proteins, some of which are uniquely present in injured cell populations in vitro, including a ~250 kDa protein common to all injured cell populations tested (Fig. 2b). FITC-Comb1 also binds proteins of ~100 and ~67 kDa in injured HaCaT and HMVEC that are not present in injured HFF, as well as proteins in confluent and post-injury HFF that do not appear in the other cell populations.

### Comb1 and UN3 induce multiple tissue repair pathways

In parallel, to determine the molecular underpinnings of the Comb1- and UN3-driven wound-healing responses, we employed quantitative real-time PCR (qPCR) to analyze mRNA expression of mediators of angiogenesis, stem and endothelial progenitor cell chemotaxis, fibroblast activation and dermal ECM remodeling, and epithelialization within granulation tissue, hypodermis, and the dermal and epidermal compartments immediately adjacent to the cutaneous injury.

#### Comb1 and UN3 increase steady-state mRNA levels of pro-angiogenic effectors after injury

**Fig. 3:**
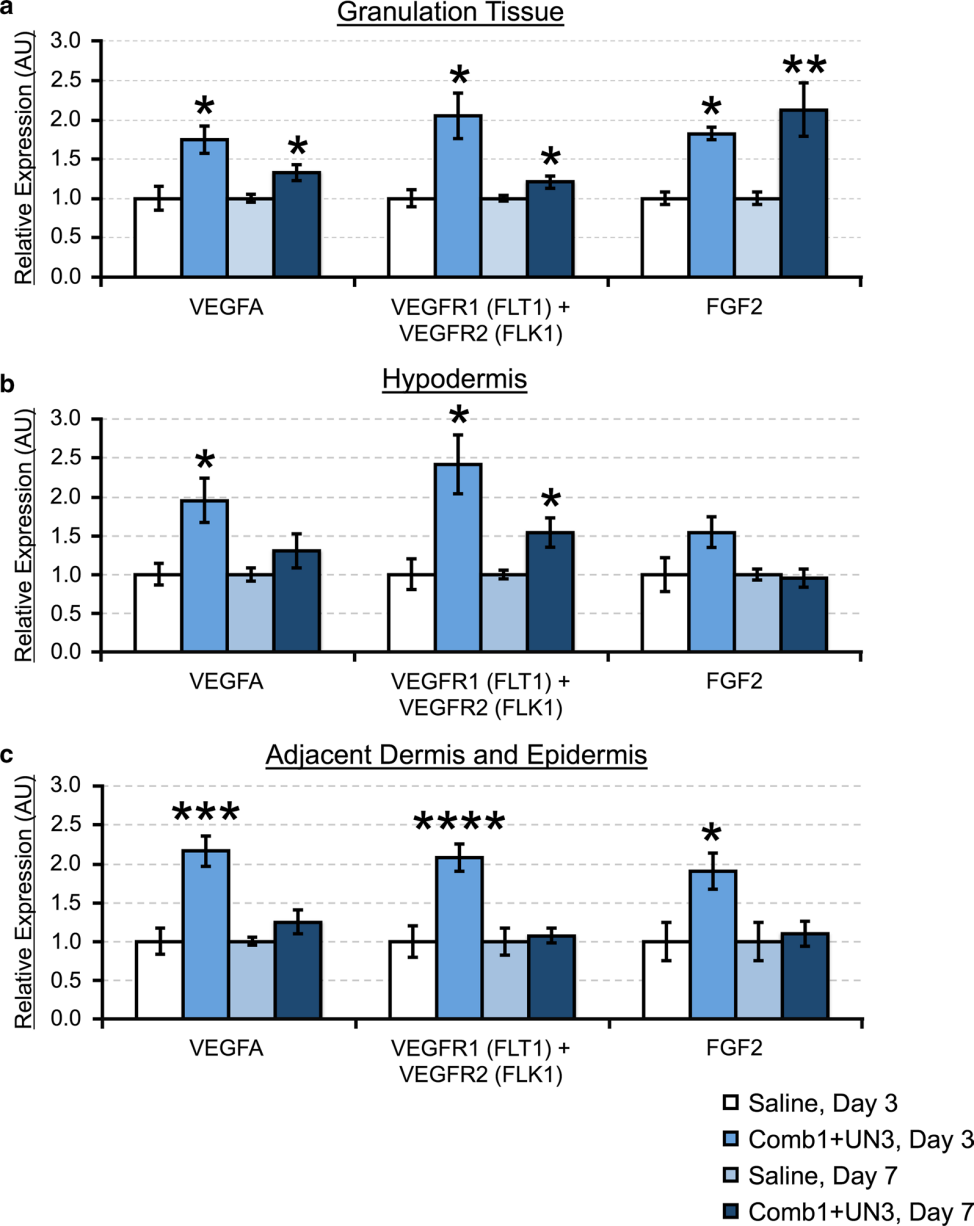
Comb1 and UN3 promote mRNA expression of key angiogenic mediators. Sample cDNAs (equivalent of 5 ng mRNA) reverse transcribed from granulation tissue (**a**), hypodermis (**b**), and portions of adjacent dermis and epidermis (**c**) were used as template, and each sample was normalized using β-actin (granulation tissue) or RPL32 (hypodermis, adjacent dermal and epidermal tissues) as a reference gene. All expression levels were calculated using the 2^−∆∆Ct^ method of relative quantitation. All data are expressed as relative abundance over saline-treated controls, ± SEM. *p ≤ 0.05, **p ≤ 0.01, ***p ≤ 0.005, ****p ≤ 0.001. Saline day 3, n = 4; Comb1 + UN3 day 3, n = 10; Saline day 7, n = 16; Comb1 + UN3 day 7, n = 19

In granulation tissue (Fig. 3a), we observe significant increases in VEGFA (80 %) and FGF2 (80 %) expression following 3 days of peptide treatment, compared to controls. After 7 days of peptide treatment, VEGFA remains significantly elevated over saline-treated controls (30 %), and FGF2 continues to significantly increase (2.1-fold). Analysis of combined VEGFR1/2 expression reveals a 2.1-fold increase in expression at day 3, and a 20 % increase in mRNA steady state expression at day 7, similar to VEGFA trends.

In hypodermal tissues (Fig. 3b), 3 days of peptide treatment induces a twofold increase in VEGFA, which remains elevated at day 7. Combined VEGFR1/2 expression levels are significantly elevated (2.4-fold) over saline controls at day 3 and day 7 (50 %). FGF2 is elevated over saline controls on day 3 by 50 % (p = 0.11), while we observe no difference in hypodermal FGF2 expression at day 7. The dermal and epidermal tissues immediately adjacent to the wound display significantly increased VEGFA (2.2-fold), VEGFR1/2 (2.1-fold) and FGF2 (90 %) following 3 days of peptide administration (Fig. 3c). However, by day 7, we detect a statistically insignificant elevation in VEGFA (30 %, p = 0.17), and observe no differences in VEGFR1/2 or FGF2 expression compared to saline treatment.

#### Comb1 and UN3 stimulate the SDF1α-CXCR4 chemotactic axis

**Fig. 4:**
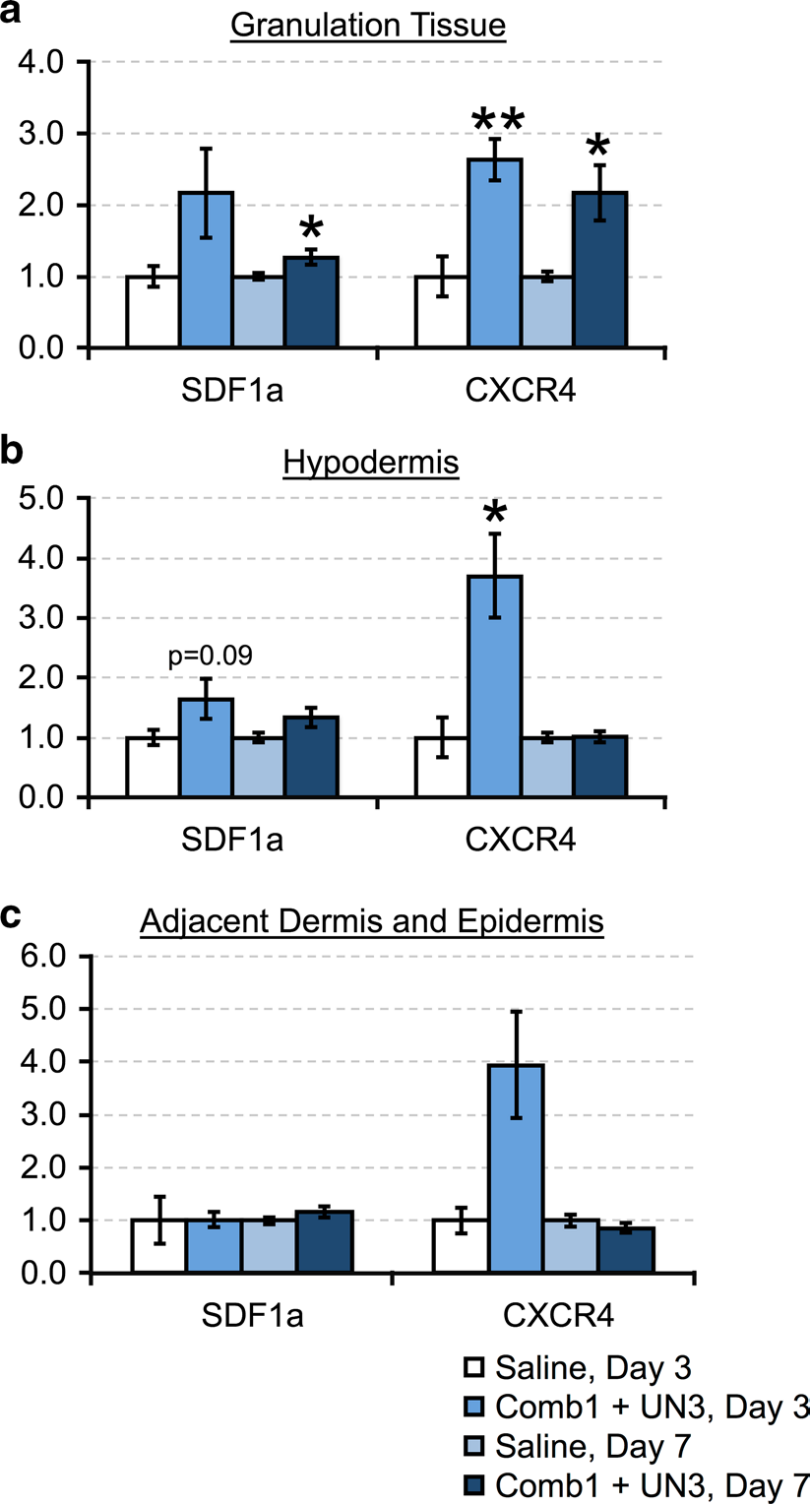
Comb1 and UN3 induce SDF1α and CXCR4 mRNA expression. Sample cDNAs (equivalent of 5 ng mRNA) reverse transcribed from granulation tissue (**a**), hypodermis (**b**), and portions of adjacent dermis and epidermis (**c**) were used as template, and each sample was normalized using β-actin (granulation tissue) or RPL32 (hypodermis, adjacent dermal and epidermal tissues) as a reference gene. All expression levels were calculated using the 2^−∆∆Ct^ method of relative quantitation. All data are expressed as relative abundance over saline-treated controls, ±SEM. *p ≤ 0.05, **p ≤ 0.01. Saline day 3, n = 4; Comb1 + UN3 day 3, n = 10; Saline day 7, n = 16; Comb1 + UN3 day 7, n = 19

SDF1α and its receptor, CXCR4, comprise a highly conserved system that regulates bone marrow and peripheral stem cell mobilization to injury sites, including endothelial progenitor cells [17], and local SDF1α elevations have been linked to improved healing in diabetic wounds [18, 19]. Given the roles of endothelial progenitor homing and differentiation in wound vascularization downstream of SDF1α, we asked whether Comb1 and UN3 impact SDF1α and CXCR4 expression in diabetic swine wounds. Peptide treatment increases SDF1α mRNA in granulation tissue and the hypodermis, and increases CXCR4 mRNA in all tissue compartments after 3 days of treatment (Fig. 4a–c). After 7 days of peptide treatment, granulation tissue SDF1α and CXCR4 mRNA remain significantly elevated over controls, while we detect no differences in these factors in either the hypodermis or the adjacent unwounded compartment.

#### Comb1 and UN3 enhance ECM-remodeling enzyme mRNA expression

**Fig. 5:**
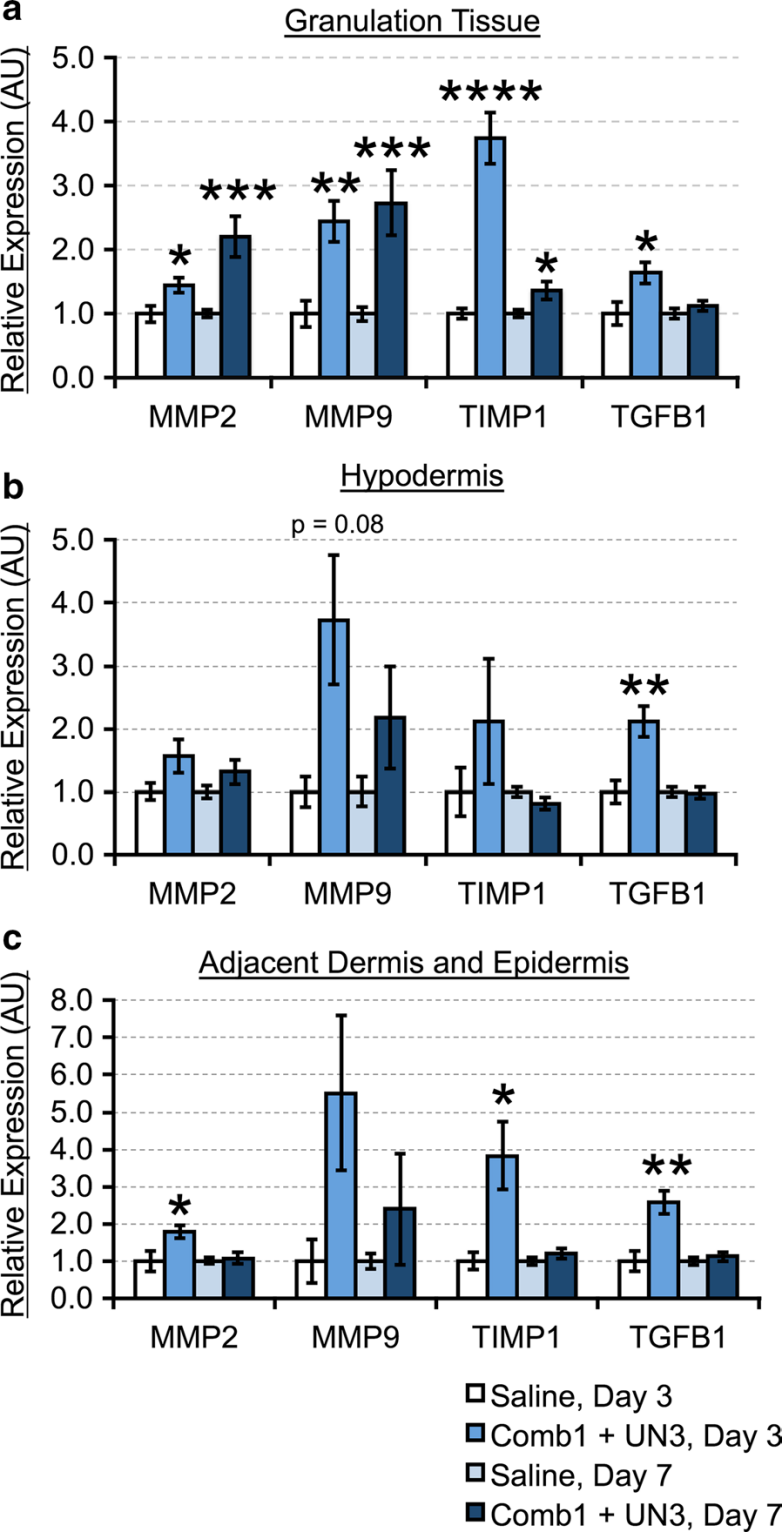
Comb1 and UN3 stimulate balanced inductions in dermal matrix remodeling factors. Sample cDNAs (equivalent of 5 ng mRNA) reverse transcribed from granulation tissue (**a**), hypodermis (**b**), and portions of adjacent dermis and epidermis (**c**) were used as template, and each sample was normalized using β-actin (granulation tissue) or RPL32 (hypodermis, adjacent dermal and epidermal tissues) as a reference gene. All expression levels were calculated using the 2^−∆∆Ct^ method of relative quantitation. All data are expressed as relative abundance over saline-treated controls, ±SEM. *p ≤ 0.05, **p ≤ 0.01, ***p ≤ 0.005, ****p ≤ 0.001. Saline day 3, n = 4; Comb1 + UN3 day 3, n = 10; Saline day 7, n = 16; Comb1 + UN3 day 7, n = 19

Peptide treatment significantly increases granulation tissue MMP2 (1.5-fold), MMP9 (2.4-fold), and TIMP1 mRNA (3.7-fold) after 3 days of treatment, compared to saline (Fig. 5a); these peptide-mediated inductions persist through 7 days of treatment (2.2, 2.7, and 1.4-fold, respectively). While we observe similar elevations in hypodermal MMP2 (1.6-fold), MMP9 (3.7-fold, p = 0.08), and TIMP1 (2.1-fold) after 3 days of treatment (Fig. 5b), these changes are not statistically significant. Within the adjacent dermal and epidermal compartments (Fig. 5c), we detect significant elevations in MMP2 (1.8-fold) and TIMP1 (3.8-fold) after 3 days of treatment, compared to saline; while peptide treatment increases MMP9 expression by 5.5-fold, this change is not statistically significant (p = 0.16). The peptide-associated increases in MMP9 mRNA in the hypodermis and the adjacent uninjured dermal compartment after 7 days of peptide treatment are not statistically significant, and we observe little difference in MMP2 or TIMP1. We also investigated transforming growth factor-β1 (TGF-β1) mRNA expression, as a major regulator of fibroblast activation and ECM synthesis, among other functions. TGF-β1 mRNA is significantly increased after 3 days of peptide treatment in all wound compartments analyzed. However, this induction is transient, as we observe no difference in TGF-β1 expression after 7 days of treatment. Thus, Comb1 and UN3 induce MMP and TIMP expression, and stimulate early TGF-β1 mRNA expression.

#### Comb1 and UN3 drive mRNA expression of keratinocyte signaling pathways

**Fig. 6:**
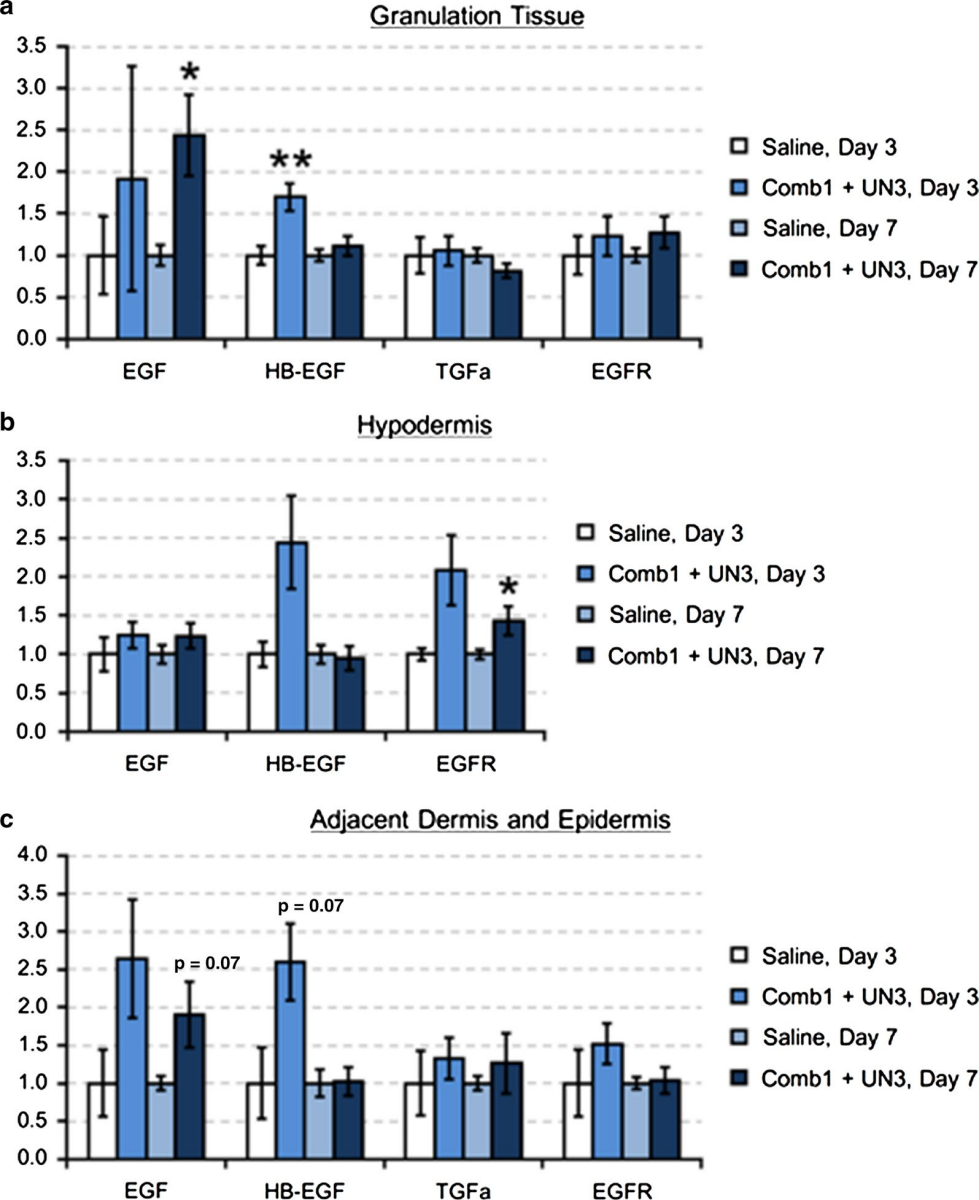
Comb1 and UN3 modulate expression of specific EGF family members to achieve re-epithelialization. Sample cDNAs (equivalent of 5 ng mRNA) reverse transcribed from granulation tissue (**a**), hypodermis (**b**), and portions of adjacent dermis and epidermis (**c**) were used as template, and each sample was normalized using β-actin (granulation tissue) or RPL32 (hypodermis, adjacent dermal and epidermal tissues) as a reference gene. All expression levels were calculated using the 2^−∆∆Ct^ method of relative quantitation. All data are expressed as relative abundance over saline-treated controls, ±SEM. *p ≤ 0.05, **p ≤ 0.01. Saline day 3, n = 4; Comb1 + UN3 day 3, n = 10; Saline day 7, n = 16; Comb1 + UN3 day 7, n = 19

In granulation tissue, 3 days of peptide treatment yields a 70 % increase HB-EGF mRNA, and EGF mRNA is significantly elevated (2.4-fold) after 7 days of treatment (Fig. 6a). While peptide treatment increases granulation tissue EGFR mRNA on day 3 and day 7 (20 and 30 %, respectively), these changes are not statistically significant. In the hypodermis, Comb1 and UN3 increase HB-EGF mRNA after 3 days of treatment, and EGFR mRNA after 7 days of treatment (Fig. 6b). In the adjacent dermal and epidermal tissues (Fig. 6c), we detect increases in EGF (2.6-fold, p = 0.18) and HB-EGF (2.6-fold, p = 0.07) after 3 days of peptide treatment, and a 90 % increase in EGF mRNA (p = 0.07) after 7 days of treatment. We observe a 50 % increase in EGFR expression on day 3, but no change in EGFR mRNA on day 7 in this compartment. We detect no difference in TGF-αmRNA expression between peptide- and saline-treated wounds in any of the tissue portions analyzed, and no TGF-α mRNA in hypodermal tissues. These data suggest Comb1 and UN3 induce specific EGF members in a time- and tissue compartment-dependent manner.

## Discussion

DFU are a leading cause of limb amputations [20], yet there is a limited arsenal of FDA-approved medications that effectively induce angiogenesis and achieve total wound closure. Indeed, a 2015 Cochrane systematic review [21] determined there is insufficient evidence to either support or refute using becaplermin and other recombinant growth factors as standard treatments for DFU. In our efforts to create advanced wound-healing therapeutics to meet the rising clinical needs, our lab created two bioactive peptides, Comb1 and UN3, that foster complete wound closure in pre-clinical murine models of cyclophosphamide-dependent healing impairments [13]. Thus, we hypothesized these two peptides would stimulate healing responses in a pre-clinical model of deficient healing in diabetes. Our present study confirms this hypothesis and extends our previous results, as Comb1 and UN3 markedly enhance wound-healing angiogenesis and re-epithelialization in full-thickness cutaneous wounds on the backs of diabetic swine. Consistent with these observations, qPCR analyses reveal that Comb1 and UN3 increase mRNA expression of growth factors and cytokines involved in angiogenesis, progenitor cell mobilization, epithelialization, fibroblast activation, and enzymes regulating ECM remodeling, lending insights into the peptides’ mechanisms of action.

### Comb1 and UN3 induce balanced MMP/TIMP expression in diabetic porcine wounds

Chronic wounds exhibit depressed TIMP expression concomitant with MMP and neutrophil elastase levels 50–100 times greater than those that heal acutely [6, 22]. This profound imbalance in the protease-to-inhibitor ratio radically disrupts ECM turnover and degrades growth factors, thus undermining the dynamic reciprocity between cells and their microenvironment required for wound resolution [7, 8, 23]. Further, the chronic wound protease burden may reduce the efficacy of growth factor-based therapeutics, including becaplermin [9, 24]. Accordingly, treatment paradigms aimed at correcting the protease imbalance have emerged as promising tactics, including therapeutics that normalize the protease-to-inhibitor ratio [25], and rationally designed molecular medicines that either resist MMP- and neutrophil elastase-dependent cleavage, or maintain their bioactivity after proteolysis.

Protease overabundance is widely considered a principal factor underlying wound chronicity [6]. Importantly, Comb1 and UN3 treatment not only promotes wound healing but also significantly enhances MMP2, MMP9, and TIMP1 mRNA expression (Fig. 5a–c). Inducing these factors, together, may aid in restoring the physiologic MMP-TIMP balance, yielding regulated matrix remodeling and preventing degradation of growth factors and their cognate receptors that enable downstream angiogenesis and re-epithelialization. Clinical assessment of wound proteases and inhibitors may represent a major advance in monitoring wound resolution, in addition to identifying mechanisms of chronicity. Indeed, Serena, et al. [26] recently developed methods to correlate global wound protease activity with healing status. At the same time, because chronic wounds may possess therapeutically responsive regions in addition to stalled, healing-incompetent areas [27], quantitative molecular profiling of local MMP/TIMP ratios through advanced transcriptional analyses could serve as a rapid and powerful diagnostic to precisely delineate margins between healing and non-healing tissues, direct treatment, and monitor healing progress. Future studies are needed to identify the range of regional MMP/TIMP mRNA ratios that might best determine healing outcomes.

In addition to stimulating MMP and TIMP mRNA expression, in silico protease substrate analyses using PROSPER and CleavPredict, online bioinformatics tools that predict protease targets [28, 29], suggest neither Comb1 nor UN3 are susceptible to MMP-mediated degradation. And, while ExPASY PeptideCutter [30] reveals neutrophil elastase recognition sites in Comb1 and UN3, the products arising from such cleavages (Table 1) are nearly identical to the individual bioactive peptides that were combined to create these entities [12, 13]. Hence, these peptides may be uniquely suited to foster healing despite the protease-rich chronic wound microenvironment, and it may be important to determine the in vivo efficacy of the original peptides used to create the combinatorial peptides tested in our present study.

**Table 1:** Bioactive matrix and PRP-derived peptide sequences and predicted cleavage product**s**

Peptide	Original sequence	Predicted products of elastase-dependent cleavage
Comb1	DINECEIGAPAGEETEVTVEGLEPG	DINECEIGA GEETEVTVEGLEPG GEETEV EGLEPG
UN3	ELLESYIDGRPTATSEYQTFFNPR	ELLESYIDGRPTA TSEYQTFFNPR

Intriguingly, neutrophil elastase is also predicted to liberate a fragment from the Comb1 C-terminal (EGLEPG) corresponding to a broadly bioactive xGxxPG consensus sequence first identified in elastin degradation products [31]. Peptides matching this motif, including VGVAPG and EGFEPG, not only regulate MMP and TIMP expression in endothelial cells, dermal fibroblasts, and macrophages, but also stimulate migratory, proliferative, and morphogenic behaviors in these cells, as well as in keratinocytes [32–35]. Additionally, VGVAPG enhances CXCR4/SDF1α mRNA and protein expression in melanoma cell lines [36], further highlighting the chemotactic and potential immunomodulatory nature of such peptides. In vivo, xGxxPG peptides stimulate angiogenesis [32], and Attia-Vigneau, et al. [37] demonstrated that a “multi-headed” synthetic peptide containing three VGVAPG repeats promotes regeneration in cultured human dermal explants. Curiously, PRP, the source of the fragments constituting UN3, is also known to induce MMPs as well as TIMPs in vitro and in vivo [38–40]. These reports, together with our data, suggest multiple mechanisms through which xGxxPG-containing and PRP-derived peptides may regulate tissue repair, including protease/inhibitor modulation and progenitor cell chemotaxis, and to our knowledge this is the first report of such peptides stimulating healing in a large animal model of diabetic wounds.

### Multiple cells and receptors may mediate Comb1 bioactivity

As several different cell populations bind and transduce signals from elastin-derived peptides such as EGLEPG, notably, Comb1 localizes to keratinocytes, fibroblasts and vascular cells in areas of swine wounds undergoing active remodeling and non-migratory cells several millimeters distal to the site of injury. Further, HMVEC, HaCaT and HFF display similarities in the molecular weights of protein effectors that interact with Comb1 at baseline and during injury repair. The presence of ~250 and ~67 kD proteins cross-linked with FITC-Comb1 in all cells tested suggests there may be several, common receptors for this peptide across cell lineages. Indeed, vascular endothelial cells, fibroblasts, keratinocytes, and immune cells respond to elastin-derived peptides through multiple receptors, including the elastin binding protein complex, cell surface-associated galectin-3, and integrin αvβ3, all of which regulate cell migration, proliferation and angiogenesis [32, 36, 41]; additionally, as Comb1 is composed of fibrillin and tenascin X fragments, it is possible that this peptide ligates with several receptors that interact with these ‘parent’ matrix molecules. Moreover, injury-dependent alterations in cell-specific receptor profiles may account for the observed differences in Comb1 binding patterns. For example, whereas post-injury endothelial integrin αvβ3 expression supports angiogenesis via adhesion to fibrin in the hemostatic plug, the absence of αvβ3 in keratinocytes, coupled with αvβ6 emergence and β1 subunit relocalization, contribute to their specific post-injury migration patterns [7]. Accordingly, advanced wound-healing therapeutics may be rationally designed to selectively activate injury-upregulated receptors, in order to potentiate cell migration, proliferation and angiogenesis and limit off-target effects. By extension, antagonists of these receptors might restrict such processes in numerous pathologies, including aberrant neovascularization in proliferative diabetic retinopathy and cancers, as well as proteolytic and migratory cascades involved in tumor metastasis. Hence, our future experiments will aim to identify and characterize the functions of the putative Comb1 receptors.

## Conclusions

In summary, Comb1 and UN3 promote wound resolution in a pre-clinical, large animal model of impaired diabetic healing, supporting results obtained previously in our cell- and rodent-based studies [12, 13]. These peptides increase steady-state mRNA levels of factors that drive angiogenesis, epithelialization, progenitor and immune cell chemotaxis, and ECM turnover in vivo, critically including balanced MMP and TIMP induction that may support tissue repair. Additionally, our findings suggest Comb1 and UN3 may have novel roles in fibroblast activation and immune cell function, which may be promising areas of future exploration. As peptide-dependent vascularization and wound closure are perhaps attributable to signals transduced through multiple, injury-regulated receptors expressed on endothelial cells, keratinocytes, and fibroblasts, discerning the precise mechanisms of Comb1 and UN3 bioactivity remain areas of ongoing investigation.

## Abbreviations

CXCR4: C-X-C chemokine receptor 4
DFU: diabetic foot ulcer
ECM: extracellular matrix
EGF: epidermal growth factor
EGFR: epidermal growth factor receptor
FGF2: fibroblast growth factor 2
FITC: fluorescein isothiocyanate
HaCaT: human adult skin keratinocytes propagated under low Ca^2+^ conditions and elevated temperature
HB-EGF: heparin-binding epidermal growth factor-like
HFF: human foreskin fibroblast
HMVEC: human microvascular endothelial cell
MMP: matrix metalloproteinase
SDF1α: stromal cell-derived factor 1α
TGF-α: transforming growth factor-α
TGF-β1: transforming growth factor-β1
TIMP: tissue inhibitor of metalloproteinases
VEGFA: vascular endothelial growth factor-A
VEGFR: vascular endothelial growth factor receptor
